# Validation of an HPLC Analytical Method for the Quantitative/Qualitative Determination of Fluticasone Propionate in Inhalation Particles on Several Matrices

**DOI:** 10.3797/scipharm.1404-11

**Published:** 2014-06-16

**Authors:** André R. Sá Couto, Daniela Espinha Cardoso, Helena Maria Cabral-Marques

**Affiliations:** Research Institute for Medicines and Pharmaceutical Sciences (iMed.UL), Faculty of Pharmacy, University of Lisbon. Av. Prof. Gama Pinto, 1649-003 Lisbon, Portugal.

**Keywords:** HPLC, Fluticasone propionate, Gamma-cyclodextrin, Analytical validation, Inhalation

## Abstract

Fluticasone propionate is a highly potent corticosteroid used to treat asthma and allergic rhinitis. It is a very effective drug, but has the inconvenient factor of being insoluble in water. Cyclodextrins were used to improve this limitation because of their ability to form inclusion complexes with guest drug molecules as well as increase the stability and bioavailability of the drugs. A rapid and simple HPLC method was developed to detect and quantify fluticasone propionate in inhalation particles on several matrices. Liquid chromatography with a UV detector at a wavelength of 236 nm, using a C18 column, was employed in this study. Isocratic elution was employed using a mixture of acetonitrile and water (60:40, v/v). The analytical method validation was performed in accordance with ICH guidelines, which included selectivity, range, linearity, accuracy, detection limit, quantitation limit, precision, robustness, and stability of solutions. This method showed to be selective and specific. Acceptable assay precision and accuracy (100 ± 5.0%) were obtained at 50– 150% of the analytical concentration of fluticasone propionate at the target concentration of 0.060 mg/mL, and good linearity (0.9958) was achieved over a range of 0.03 to 0.09 mg/mL for fluticasone propionate. The proposed HPLC method proved to be reliable. The validation and application of this method can be adopted for determining the fluticasone propionate in: assays, impingers and impactors, diffusion cells, dissolutions, and other tests. In addition, this method can be adapted and used in the pharmaceutical industry for routine analysis.

## Introduction

Chronic respiratory diseases, which include asthma and chronic obstructive pulmonary diseases, comprise a major cause of death and disability for all age groups and regions in the world [1]. In the last estimates of the World Health Organization, 235 million people had asthma, 64 million suffered from chronic obstructive pulmonary disease, and a few other million had allergic rhinitis or other often underdiagnosed respiratory diseases [2].

Traditionally, the treatment of these diseases consists of treatment with inhalation drugs (such as corticosteroids and (β-adrenergic agonists) formulated to be used as a nasal spray, dry powder inhaler, metered-dose inhaler, or as a nebulizer [1]. Even though some commercial formulations of inhaled microparticles can only deliver a low percentage (10-20%) of their labelled dose in the interest area of the lungs [3], these devices and their formulations are still effective, non-invasive, and convenient ways to provide a pulmonary administration of small molecule drugs and biopharmaceuticals [4].

Lung delivery systems are advantageous and an alternative to systemic drug delivery because they allow the topical action of drugs [5], by providing a targeted therapy on the affected airway’s area with a higher drug concentration, but at the same time with reduced systemic side effects derived from the lower systemic exposure to the drugs [4].

**Fig. 1.**
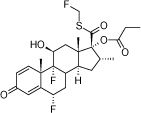
Chemical structure of fluticasone propionate [1]

One of the drug classes used in lung therapy is corticosteroids, very important in the regulation of the inflammatory process and response. Fluticasone propionate (FP), a second-generation trifluorinated glucocorticosteroid based on the androstane nucleus, is a highly potent drug and one of the most used corticosteroids to treat asthma (inhalation) and allergic rhinitis (intranasally) [1]. FP (Figure 1) is a very effective drug in the treatment of these diseases, but has a limitation due to its water insolubility that may lead to a reduction in the local absorption of the drug [6]. In order to be pharmacologically active, drugs should be lipophilic to permeate the biological membrane (through passive diffusion) and at the same time must have some aqueous solubility [7].

To exceed this and other limitations of these formulations, cyclodextrins (CyDs) may be used. CyDs are oligosaccharides composed of glucose units connected by a a-1,4-glycosidic linkage with a truncated cone structure. They have been increasingly used as pharmaceutical excipients because of their ability to form inclusion complexes with guest drug molecules, due to their relatively apolar inner cavity [5, 6].

In addition, by complex formation, the CyDs also increase drug stability and bioavailability [8, 9] leading to an improvement in the local effect of these molecules [10]. CyDs can also solve other disadvantages of the inhaled therapy - the short duration of drug action - by developing controlled release formulations.

It is very important to use analytical methods validated for the quantification of drugs. The FP quantification has already been described in the literature: pharmacopoeial methods [11, 12] or other published methods [13, 14], but these methods use organic solvents in the preparation of the sample, such as acetonitrile and methanol.

However, in the present case, the sample is a complex of FP with CyDs and as the latter precipitates with those solvents, it is impossible to use those methods to quantify FP, being necessary to use a solvent able to solubilize both components.

Thus, the aim of this work was to develop and validate an HPLC method that could be rapid, simple, and at the same time versatile enough to detect and quantify FP on inhalation particles on several matrices, and possible applications such as: assay, aerodynamic assessment of fine particles (impinger and impactor), *in vitro* release (diffusion cells), and dissolution tests.

## Results and Discussion

### Validation Parameters

#### Selectivity (Stability-Indicating Evaluation)

**Fig. 2.**
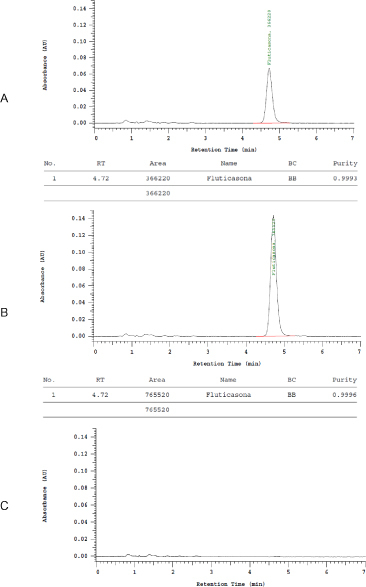
Selectivity parameter of the analytical method proposed A: Sample: γ-CyD/FP complex + PVA 0.1%; B: Standard solution of FP; C: Placebo solution

The analytical method was demonstrated to be selective and specific for the intended purposes. The chromatograms (Figure 2) show that there is no interference of the solvent, mobile phase, and excipients with spiked FP. In addition, for the cases except the placebo solution (without FP), FP showed excellent peak purity (0.999).

#### Linearity and Range

The linearity between the peak area and the concentration was examined (Table 1). Results have shown that the method is linear over the specified range with a correlation factor of 0.9958.

#### Limit of Detection (LOD) and Limit of Quantitation (LOQ)

The LOD and LOQ were found to be 0.0067 mg/mL and 0.0203 mg/mL, respectively. The %RSD of the six solutions of FP used to confirm the LOQ value was 5.9%. This value showed that this parameter was validated.

**Tab. 1.** Linearity from standard solutions in the range 50–150% of the nominal standard concentration 0.060 mg/mL

Level	Concentration (mg/mL)	Peak area(mAU)	Response factor
	(X)	(Y)	(Y/X)
L50%_1	0.0303	690.4936	22826.24
L50%_2	0.0303	663.6248	21938.01
L50%_3	0.0300	531.5904	17719.68
L80%_1	0.0484	1085.7753	22433.37
L80%_2	0.0484	1066.7892	22041.10
L80%_3	0.0480	1053.8915	21956.07
L100%_1	0.0605	1323.7201	21879.67
L100%_2	0.0605	1298.9583	21470.38
L100%_3	0.0600	1289.1318	21485.53
L120%_1	0.0726	1624.1429	22371.11
L120%_2	0.0726	1620.4866	22320.75
L120%_3	0.0720	1612.9238	22401.72
L150%_1	0.0908	2077.4456	22891.96
L150%_2	0.0908	2034.7564	22421.56
L150%_3	0.0900	1920.1162	21334.62

Equation on chart: y = 22909x - 55.918

Slope: 46.4538

r^2^: 0.9916

r: 0.9958

Residual standard deviation: 22908.97

#### Accuracy (% Recovery)

Results have shown that the mean recovery of FP is within 100 ± 5.0% for the three concentration levels (Table 2). Specifically, the mean recovery was found to be 99.9% for 0.030 mg/mL, 101.6% for 0.060 mg/mL, and 99.9% for 0.015 mg/mL.

**Tab. 2.** Accuracy at three concentration levels covering the range of 50% to 150% from the nominal standard concentration, 0.060 mg/mL

Level	Weight (mg)	Volume (mL)	Dilution	C (mg/mL)		Recovery %	Average ± % RSD
				(Real)	(Recovery)		
50%_1	12.5	20	0.05	0.0313	0.0301	96	
50%_2	12.5	20	0.05	0.0313	0.0329	105	99.9%
50%_3	12.0	20	0.05	0.0300	0.0294	98	± 4.8%
100%_1	12.5	20	0.1	0.0625	0.0648	104	101.6%
100%_2	12.5	20	0.1	0.0625	0.0653	104	± 4.2%
100%_3	12.0	20	0.1	0.0600	0.0580	97	
150%_1	12.5	20	0.15	0.0938	0.0905	96	99.9%
150%_2	12.5	20	0.15	0.0938	0.0988	105	± 4.9%
150%_3	12.0	20	0.15	0.0900	0.0879	98	

#### Precision

This was obtained by the investigation of repeatability, system precision, and intermediate precision (Table 3). System precision was found to be less than 2.0% (between the five injections). Furthermore, the peak asymmetry and efficiency of the chromatographic column were analyzed, proving to be in compliance with the established acceptance criteria. The repeatability of the method was found to be 3.7% for *γ*-CyD:FP+ PVA 0.1% complex and 4.0% for *γ*-CyD:FP+ PEG 0.5% complex (six samples each). For intermediate precision, the results obtained were 8.3% for the powder with PVA 0.1% and 5.2% for the powder with PEG 0.5%.

**Tab. 3.** Repeatability and intermediate precision which compose the precision parameter of the analytical method proposed

Sample	Repeatability	Intermediate Precision	Repeatability	Intermediate Precision
	γ-CyD : FP complex + PVA 0.1% (mg/ml)		γ-CyD : FP complex + PEG 0.5% (mg/ml)	
1	0.0684	0.0684	0.0601	0.0601
2	0.0719	0.0719	0.0622	0.0622
3	0.0690	0.0690	0.0628	0.0628
4	0.0707	0.0707	0.0653	0.0653
5	0.0645	0.0645	0.0581	0.0581
6	0.0678	0.0678	0.0621	0.0621
7		0.0766		0.0669
8		0.0817		0.0647
9		0.0759		0.0703
10		0.0818		0.0683
11		0.0641		0.0653
12		0.0740		0.0616
Average	0.0687	0.0722	0.0618	0.0640
%RSD	3.72	8.25	3.98	5.22

Samples 1–6 were performed by analyst 1 in Day 1 and samples 7–12 were performed by analyst 2 in Day 2.

#### Stability of FP Standard Solution

By the analysis of the % recovery (Table 4), it was found that both standards were stable at the end of this period of time. The standard stored at room temperature varied between 99-102% and the refrigerated standard varied between 98-103%.

**Tab. 4.** Stability of FP solutions either stored at room temperature (25°C) and refrigerator (2 to 8°C) for a week

Standard solution	% Recovery
Room temperature (25°C)	99-102%
Refrigerator (2 to 8°C)	98-103%

#### Robustness

As shown in Table 5, for the pore size of the membrane filter, the %RSD between peak areas less than 2% was obtained showing that the sample solutions can be filtered through a pore size of 0.45 or 0.22 μm. The change in the mobile phase did not show any impact on the peak areas for both the standard and sample, as the results obtained presented a %RSD of about 2.0%. However, the variation of the flow rate showed a %RSD greater than 2.0%, indicating that measurements are susceptible to variations in analytical conditions. Therefore, the flow rate should be suitably controlled for the reliability of the analysis.

**Tab. 5.** Robustness of the analytical conditions of the method proposed

Analytical Condition	Sample (Peak area)	Analytical Condition	Standard (Peak area)	Sample (Peak area)	Analytical Condition	Standard (Peak area)	Sample (Peak area)
Filter 0.45 μm	2776.97	1.0 mL/min	1432.41	2776.97	60:40 (ACN:H_2_O)	1432.41	2776.97
	2818.04		1481.54	2818.04		1481.54	2818.04
	2738.04		1428.25	2738.04		1428.25	2738.04
Filter 0.22 μm	2726.63	0.9 mL/min	1692.02	3024.37	55:45 (ACN:H20)	1405.43	2821.53
	2775.86		1624.12	3031.55		1482.50	2835.31
	2775.86		1592.80	3105.28		1454.17	2927.59
		1.1 mL/min	1278.88	2483.92	65:35 (ACN:H_2_O)	1422.57	2773.01
			1277.86	2498.06		1454.11	2756.51
			1304.08	2585.06		1452.64	2781.15
Average	2761.88	Average	1456.88	2809.53	Average	1445.96	2803.13
%RSD	1.26	%RSD	10.6	8.32	%RSD	1.81	2.02

## Experimental

### Chemicals

Acetonitrile super gradient HPLC and ethanol 96% v/v were obtained from VWR (Fontnay-Sous-Bois, France). Polyvinyl alcohol 4-88 (PVA) and polyethyleneglycol (PEG) 6000 were obtained from Merck (Darmstadt, Germany). *γ*-Cyclodextrin (*γ*-CyD) (Cavamax® W8 Pharma Gammadex) and FP were kind gifts from Wacker (Burghausen, Germany) and Hovione Farmaciência S.A. (Loures, Portugal), respectively.

### Apparatus

The Merck Hitachi Lachrome Elite HPLC system (Japan) with an L-7100 pump, an L-7200 autosampler, L-7350 column oven, L-7450A DAD detector, and Ezochrom Elite software was employed for the selectivity test and Agilent Technologies 1200 series HPLC system with a G1310A isocratic pump, G1314A UV detector, and G1329A autosampler was used for all other tests. The LiChrospher 100 RP18 column (5 μm, 125 mm length, 4.0 mm inner diameter) was utilized in this study and it is from Merck (Darmstadt, Germany). The C18 column with 5 μm, 125 mm length, 4.0 mm inner diameter was kept at room temperature. A mixture of acetonitrile and water (60:40, v/v) was used as the mobile phase at a flow rate of 1.0 mL/min. The UV detector was regulated for 236 nm and the injection volume w 10 μL.

### Preparation of the y-cyclodextrin/Fluticasone Propionate (y-CyD:FP) Complexes

The complexes were prepared by agitation of an aqueous solution of y-CyD (14 mg/mL) with the FP (6 mg/mL). A polymer was added to help the complexation process (PVA 0.1% and PEG 0.5%). These dispersions were submitted to the spray drying process in order get a powder.

### FP Standard and Sample Solution Preparation

A stock standard solution was prepared by dissolving 12 mg of FP in 20 mL of ethanol to obtain a solution with a known concentration of 0.6 mg/mL. A nominal standard solution was prepared by diluting 1 mL of stock standard solution to 10 mL with the same solvent to obtain a solution with a known FP concentration of 0.06 mg/mL. The sample solutions were prepared from two different inhalation powders containing complexes of y-CyD:FP and polymer, either PVA 0.1% or PEG 0.5%. The powders were accurately weighed in accordance with each test and diluted with ethanol.

### Validation of the Method for the Determination of FP

Analytical method validation for the determination of FP was performed in accordance with ICH guidelines [15], which include selectivity, range, linearity, accuracy, detection limit (LOD), quantification limit (LOQ), precision, robustness, and stability of solutions.

#### Selectivity

Solutions of the mobile phase, solvent, excipients, y-CyD: FP complex, and y-CyD spiked with FP were tested in order to evaluate the selectivity and specificity of the method. As acceptance criteria, the chromatographic peaks detected from the mobile phase, solvent, and excipients should not interfere in the FP chromatographic peak. For this study, the FP purity peak was also evaluated.

#### Linearity and Range

To evaluate the linearity, standard solutions covered the range between 50-150% of the nominal standard concentration (0.060 mg/mL). Thus, five solutions of FP with different concentrations (0.030, 0.048, 0.060, 0.072, and 0.090 mg/mL) were prepared by diluting different volumes of the 0.6 mg/mL stock standard solution using ethanol as a solvent. To achieve a concentration of 0.030 mg/mL, 0.5 mL of stock solution was diluted to 10.0 mL; 0.8 mL was diluted to 10.0 mL for a concentration of 0.048 mg/mL; 1.0 mL was diluted to 10.0 mL for a concentration of 0.060 mg/mL; 1.2 mL was diluted to 10.0 mL for a concentration of 0.072 mg/mL; and 1.5 mL was diluted to 10.0 mL for a concentration of 0.090 mg/mL. This test was performed in triplicate.

#### Detection Limit (LOD) and Quantification limit (LOQ)

These were obtained from the linearity test as follows:


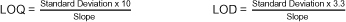


In addition, results were confirmed from six FP solutions prepared at a LOQ concentration (0.0203 mg/mL) where the relative standard deviation (%RSD) was evaluated.

#### Accuracy (% recovery)

This was assessed using nine determinations at three concentration levels covering the specified range (50% to 150%). Thus, three 10-mL volumetric flasks with y-CyD ethanolic solutions were spiked with 0.5, 1.0, and 1.5 mL of FP stock standard (0.6 mg/mL) in order to obtain a 0.14 mg/mL y-CyD final concentration and 0.030 (50%), 0.060 (100%), and 0.090 (150%) mg/mL of FP, respectively. The solutions were injected and the percentage of recovery was calculated.

#### Precision System

This was analysed from five consecutive injections of the standard solution, expressed in %RSD. Furthermore, the peak asymmetry (0.8-1.5) and efficiency of the chromatographic column (number of theoretical plates e 2000) [16] were evaluated.

#### Repeatability

The repeatability was evaluated by the variability of the assay, expressed in %RSD. Six sample solutions composed of y-CyD:FP complexes were used. In addition, two different inhaled powders containing complexes of y-CyD:FP and a polymer (either PVA 0.1% or PEG 0.5%) were analyzed.

#### Intermediate Precision

This was measured by repeating the analytical procedure under normal operating conditions being expressed as intra-laboratory variation (performed by two different analysts on two different days). Each analyst prepared six samples of the y-CyD:FP complexes (complexes formed in the presence of PVA 0.1% or PEG 0.5%) at the concentration 0.060 mg/ml on its specific day. The calculation was performed with 12 samples and the assay variability was expressed as %RSD. However, the results obtained from six samples prepared by analyst 1 were also used for the repeatability test calculation.

#### Stability of Standard Solutions

This was examined by analyzing the solutions stored at room temperature (25°C) and refrigerator (2 to 8°C) for a week. The % recovery of the peak area was calculated from the initial concentration.

#### Robustness

This was evaluated from small variations in method parameters such as mobile phase proportion (±9%) and flow rate (±10%). The pore size of the membrane filter was also tested (0.22 and 0.45 μm).

## Conclusion

A simple HPLC method for isocratic liquid chromatography has been described and validated (ICH guidelines) for the qualitative and quantitative determination of FP in inhaled particles in several matrices (CyDs complexes and polymers). It can also be used for other matrices having the advantage in relation to those from the literature of being innovative and much quicker, using a single and *greener* solvent (ethanol). The analytical method showed to be selective and specific. Acceptable assay precision (< 8% RSD) and accuracy (100 ± 5.0%) were obtained at 50-150% of the analytical concentration of FP (target concentration of 0.060 mg/mL), and good linearity (0.9958) was achieved over the range from 0.03 to 0.09 mg/mL for FP. The proposed HPLC method had a run time of 7 minutes and proved to be reliable, only requiring one parameter to be suitably controlled, the flow rate (1.0 mL/min). The validation and application of this method can be adopted for determining FP in several possible applications such as: assay, aerodynamic assessment of fine particles (impingers and impactors), *in vitro* release (diffusion cells), and dissolution tests. In addition, this analytical method has the advantage of being quite simple, versatile, and economically viable that can be easily adapted for use in the pharmaceutical industry for routine analysis.
